# Assessment of Knowledge, Attitudes, and Practices (KAP): Public Health and Economic Burden of Tuberculosis in Jarso District of West Wollega Zone, Oromia, Western Ethiopia

**DOI:** 10.1155/2022/3314725

**Published:** 2022-12-08

**Authors:** Gemechu Hailu, Monenus Etefa, Feyissa Begna

**Affiliations:** ^1^Jarso District Agricultural Office, Geba Defino, Oromia, Ethiopia; ^2^Ilu District Agricultural Office, Teji, Oromia, Ethiopia; ^3^Jimma University, College of Agriculture and Veterinary Medicine, Jimma, Oromia, Ethiopia

## Abstract

Tuberculosis is a communicable mycobacterial disease of humans and animals caused by members of *Mycobacterium tuberculosis* complex, highly impacting the public health and economy of the country in endemic areas. Retrospective and cross-sectional study was conducted between March and August 2021. For knowledge, attitude, and practices study, five villages were randomly selected using simple random sampling. To quantify the public health burden, one-year data were collected from Jarso Health Center, and unregistered patients were identified using snowball method, and the estimation was based on disability-adjusted life years. Younger age groups (18-40 years) had shown 105.8 times more odds of knowledge towards tuberculosis than the older (>60 years). Moreover, tertiary levels of education had 9395.1 times more odds of knowledge towards tuberculosis compared to illiterates. The estimated economic burden was 7,731.25US$. Of the 51 tuberculosis patients, two died from the disease, resulting in 45.03 disability-adjusted life years. Communities of the study district were heard about tuberculosis, however, unaware of the cause and sources of the infection. Therefore, further strategic and continuous community-based health education and awareness should be given for effective control and prevention of tuberculosis in the study area.

## 1. Introduction

Tuberculosis (TB) is a highly communicable and progressive granulomatous infectious disease caused by the gram-positive, acid-fast bacilli classified under the group of bacteria called *Mycobacterium tuberculosis* complex (MTBC) [1]. It signifies different species including *M. tuberculosis* and *M. bovis* [2]. *Mycobacterium tuberculosis* (MTB) primarily causes TB in humans, whereas *M. bovis* predominantly affects cattle causing bovine tuberculosis [1]. Tuberculosis causes estimated 2 million deaths annually, and the death toll is worsened by the emergence of drug-resistant *M. tuberculosis* [3]. Even though the incidence rates of TB have slowly declined since the year 2000 globally, there are still major challenges associated with TB diagnosis, treatment success rate, patient compliance, and multidrug-resistant tuberculosis (MDR-TB) [4]. Worldwide, TB continues to be responsible for the most deaths attributable to a single infectious disease [5]. In order to meet these challenges, the World Health Organization (WHO) has suggested the goal of eliminating TB by 2050 where elimination of TB is defined as less than one TB case per million populations per year [4, 6, 7].

Tuberculosis is among the major deadly diseases in human and animals in Ethiopia. Because of high livestock population and economic dependency on livestock and livestock products, there is high connection between livestock and tuberculosis transmission in the country and current study area. Because of animals providing many benefits to people, they interact with their animals in their daily lives, both at home and away from home. Humans and animals share some common risk factors for transmission of TB, such as nutrition or malnutrition, age, crowding, and extent of exposure. There are many others which are likely to be restricted to humans or animals only, such as substance abuse in humans and environmental contamination in animals [8].

Tuberculosis is transmitted from infected animals to humans through close contact and ingestion of raw animal products particularly raw or unpasteurized milk of diseased animals. Although the most commonly affected species by *M. bovis* are members of the Bovidae, even humans can be infected by the bacteria [9]. Bovine tuberculosis (BTB) is a chronic and zoonotic disease that has a huge economic loss as a result of the disease burden globally. Over 50 million cattle are estimated to be infected with *M. bovis* worldwide resulting in production loss of about 3 billion USD each year [10]. Also, it signifies financial burden due to reduction of productivity to the livestock sector that can be explained through reduction of milk production and carcass condemnation [1, 11].

Tuberculosis is the seventh most important cause of premature mortality and disability globally and is expected to remain among the 10 leading causes of death until at least 2020 [12, 13]. In 2017, there were about 45 million disability-adjusted life years (DALYs) [14] and estimated 10 million active TB cases, with an estimate of 1.5 million TB deaths worldwide in 2018 [15]. Currently, treatment is usually conducted in outpatient clinics, where health workers are exposed to infection [16]. For this reason, and due to these individuals' vulnerability, they must have considerable knowledge about the disease [17]. Most countries is aimed at providing TB diagnosis and treatment free of charge within public health services substantially over the past two decades through national efforts and global financial support [18]. However, many TB patients and families are still facing very high direct and indirect costs due to TB illness and care-seeking, hampering access and putting people at risk of financial ruin or further impoverishment [18, 19].

Zoonotic tuberculosis (TB) is a form of tuberculosis in people caused by *Mycobacterium bovis*, which belongs to the *M. tuberculosis* complex [20, 21]. It often affects sites other than the lungs (extrapulmonary), but in many cases, it is clinically indistinguishable from TB caused by *M. tuberculosis*. *Mycobacterium bovis* mainly affects cattle as well as ranges of wild animal species [22]. It results in important economic losses and trade barriers with a major impact on the livelihoods of poor and marginalized communities. In 2016, there were estimated 147,000 new cases of zoonotic TB in people globally and 12,500 deaths due to the disease [22].

The African region carries the heaviest burden, followed by the South-East Asian region [22, 23]. Different studies have indicated critical knowledge gaps and the associated risky practices towards BTB in Ethiopia. Educational efforts in Ethiopia were reserved for addressing human to human transmitted TB even though the impact of TB from animal to human is not limited [24, 25]. While children are part of the community at risk of acquiring BTB, health education remains a useful way of promoting awareness among themselves and even parents [26].

Although tuberculosis is a preventable and treatable disease, it causes more than a million deaths each year globally [27]. It is known that tuberculosis is among the top public health and economic problems in developing countries including Ethiopia. Currently, Ethiopia is ranked eighth among the 22 high TB burden countries in the world and ranks third in Africa [28]. The country has a huge livestock population in Africa, and several livestock owners of the country have direct and/or indirect contact with animals which facilitate transmission of zoonotic diseases from animals to humans and vice versa including TB. Furthermore, many people in Ethiopia have poor consumption habits of animal products and by-products which are the major risk factors of tuberculosis in animals and humans [29].

Despite the public health importance of TB, knowledge of the community toward the disease and its public health and economic burden remains poorly understood in the country. Communities of the Jarso district of West Wollega Zone also may not be out of this situation, and burdens of the disease may be high in the district. There is no previously conducted research regarding the assessment of knowledge, attitudes, and practices of people toward tuberculosis, and the burdens of the disease were not yet assessed in the Jarso District. By considering these gaps, the ultimate goal of the current study was to assess the knowledge, attitudes, and practices (KAP) of the community towards tuberculosis and to estimate public health and economic burden of the disease in Jarso District, Oromia Regional State, Western Ethiopia.

## 2. Materials and Methods

### 2.1. Study Area Descriptions

**Figure 1:**
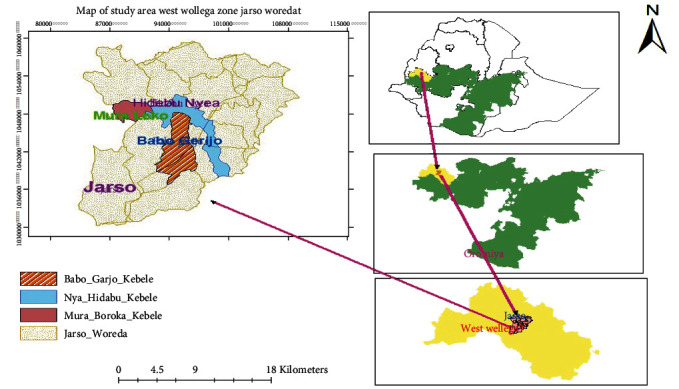
Maps of the study area.

The study was conducted in West Ethiopia, Oromia Regional State, Jarso district (Figure 1) from March 2021 to August 2021. Jarso is among the 20 rural districts and 3 urban districts of the West Wollega Zone. General description of the study area is shown in Table 1.

Livestock management practices in the district were based on the traditional knowledge of the farmers, and it was noted that the farmers lack adequate knowledge and skills in improved livestock management practices. Jarso Health Center provides services for cases that come from 16 villages in the district. Among the sixteen villages of the district, five villages (Jarso 01, Jarso 02, Babbo Gerjo, Nyeha Hidebu, and Mura Borokka) were selected by a simple random sampling method for the KAP study.

### 2.2. Study Population

For the retrospective study, all TB patients that were registered in Jarso District Health Center between September 2019 and September 2020 and unregistered patients were searched extensively using snowball sampling method. For the KAP study, 320 people older than 18 years from the five selected villages were assessed.

Inclusion and exclusion criteria were as follows: for retrospective study, all age group patients with history of tuberculosis were included in the study. For knowledge, attitudes, and practices (KAP) study, all people older than 18 years from the five selected villages were included; age groups of less than 18 years and greater than 90 years were excluded because they may not give appropriate responses.

### 2.3. Study Design

A cross-sectional study was conducted to assess the community's knowledge, attitudes, and practices (KAP) toward tuberculosis, and retrospective study design combined with snowball method was conducted to collect relevant data that helps to assess public health burden of tuberculosis. The data were collected by using pretested semistructured questionnaire.

### 2.4. Sampling Method and Sample Size Determination

For the retrospective study, sample size was determined based on the cases recorded on case books of human tuberculosis at Jarso District Health Center for a period of one year. Hence, because of limited number of TB patients registered in the health center between the study periods, all the registered patients were included in the present study. Additionally, unregistered TB patients (those who did not get treatment from the district health center but have treated anywhere else at the different healthcare centers like private clinics, hospitals in the neighboring districts, and bigger cities and known to positive for tuberculosis) were also searched by using snowball method depending on the information obtained from the registered patients from their neighbors and/or surrounding areas so that all the contacted unregistered TB patients were also involved in the study. Unregistered TB patients' data were confirmed by contacting the healthcare centers at which the patients were diagnosed. For knowledge, attitudes, and practices (KAP) study, five villages from the 16 villages of the district and primary data from participants of the study were collected by using simple random sampling technique.

The total sample size of the households was determined by using the formula recommended by Arsham [30] for the KAP analysis. That is, *N* = 0.25/(SE)^2^, where N = required sample size and SE = standard error. Depending on this formula, the total number of respondents who participated in the study was calculated by assuming the standard error of 2.8%, just to increase the number of participants, the precision level at 5%, and the confidence interval of 95%; then, 320 participants were selected from all the five villages of the study district for KAP interview.

### 2.5. Data Collection

#### 2.5.1. Retrospective Data Collection from the Health Center

Tuberculosis patients registered in Jarso District Health Center between September 2019 and September 2020 (for a period of one year) were collected. The information gathered from Jarso Health Center was patient's name, age, sex, residence (urban or rural), educational status, marital status, clinical form of tuberculosis (pulmonary or extrapulmonary), occupation, outcome of the disease (inpatient or outpatient), type of treatment given (antibiotic or other), and outcome of treatment (died or cure). Unregistered tuberculosis patients were also searched to be included in the present study. Then after, face-to-face interview was made for economic and public health burden analysis due to the disease.

#### 2.5.2. Questionnaire Survey

Participants were interviewed face-to-face at their own villages or homes using a pretested structured and semistructured questionnaire survey for KAP study. Demographic information (age, sex, residence/urban or rural, educational status, marital status, religion, occupation, form/type of TB, age at infection, whether visit hospital/health center and treated, the outcome of TB treatment (survived or died), and duration of illness until recovery or death) and other information of the respondents regarding their exposure were also covered by the questionnaire. Extensive searching of TB patients, those nonregistered in the health center, was also performed by gathering data from contacted patients whether they know of their neighbors.

To assess public health burden due to tuberculosis exposure, the data that were simultaneously collected were related to age at infection, adverse effects from treatment, and duration of illness until recovery and age at death due to the disease if the outcome of the disease was death. Moreover, the data was collected on expenditures incurred related to direct and indirect costs such as healthcare costs (treatment costs like vaccines, diagnosis, wound care costs, and antibiotics) and nonhealthcare costs (transportation, food, loss of time while seeking medical care and bed sleeping, and number of workdays lost of families to help the patient) to assess economic burden of tuberculosis. To evaluate the KAP of the community towards tuberculosis, the collected data was about source of infection, clinical signs, means of transmission from animals to humans and vice versa, its treatment, their attitudes toward public health impact of tuberculosis, and their practices concerning prevention and control of the disease particularly pasteurization of milk. The questionnaire was first developed in English and then translated to Afan Oromo language (the native language of the respondents) for appropriateness and easiness in approaching the participants.

### 2.6. Socioeconomic and Public Health Burden Assessment

To estimate the economic burden of tuberculosis, the total sum of direct and indirect costs (the sum of healthcare and nonhealthcare costs) was expressed in terms of monetary loss. The direct healthcare costs are card costs, costs of diagnosis, costs of antibiotics, costs of supplementary drugs like multivitamins, costs of antipains, and costs of vaccination; whereas, the nonhealthcare costs are costs of transportation per round trip, the opportunity to loss workdays by themselves and their families, and time losses while seeking medical treatments and costs of food and accommodation. These costs were estimated based on the responses from the study participants regarding the whole expenditures of their total costs. To compute the costs of loss of workdays, the period between stopping work until they become clinically better was considered. According to the response from TB patients, loss of workdays by themselves and their escorts varied from patient to patient based on various factors.

To estimate the public health burden of tuberculosis, the disability-adjusted life years' (DALYs) estimation method that was developed in the 1990s by the Harvard School of Public Health, the World Bank, and the World Health Organization (WHO) to describe death and loss of health due to diseases, injuries, and risk factors for all regions of the world [31] was applied. DALYs is the sum of years of life lost (YLL) due to premature death by tuberculosis and years of lived with disability (YLD) [32]. One DALY represents the loss of one year of life, and the total number of DALYs means the total burden of the disease. In general, public health and economic burden of tuberculosis were assessed based on the cost classification by Jo et al. [33]. Costs indicated as direct costs, consisting of healthcare and nonhealthcare costs, were expressed in monetary terms and indirect costs in terms of DALYs summarized as shown (Table 2).

### 2.7. Data Management and Analysis

The collected data were entered into Microsoft Office Excel version 2010, then coded and imported into Stata version 15 statistical software package (StataCorp, USA). Descriptive statistics such as frequency, percentage, or proportion were used to calculate the collected data. The KAP orders of the respondents and key predictors (age, sex and residence, educational status, and occupation) were analyzed using ordinal logistic regression model, and odds ratio was used to determine the strength of association between KAP orders and predictors. The data were analyzed using univariate and multivariate logistic regressions and odds ratios (ORs), and their corresponding confidence intervals (CIs) of 95% were calculated, and all *p* values less than 0.05 were considered as statistically significant.

The opportunity costs, losses of workdays by TB patients and by their escorts while medical treatments, were valued in monetary terms using the estimated day labor cost (70 ETB/day) according to local context. The average United States dollar (USD) and Ethiopian birr (ETB) exchange rate was estimated at 1USD to 34.95 ETB during this study [34]. YLL was calculated as the number of human deaths within the age category multiplied by the life expectancy of the concerned age category. The average Ethiopian life expectancy during the study period was 66.5 years [35]. YLD was calculated by the total period lived with disability and its corresponding disability weight. The average disability weight (DW) of TB is 0.271 [36] DALY = YLD + YLL, whereas,

(1) $$\begin{array}{c} \text{YLD}=\text{Number of cases}*\text{Duration of disability}*\text{Disability weight},\\ \text{YLL}=\text{Number of deaths}*\text{Life expectancy at the age of death}. \end{array}$$

### 2.8. Limitation of the Study

This study digged a lot about retrospective assessment of economic and public health burden of BTB encountered in one year period (only in 2019). However, it did not cover long period analysis (for example, three-year or five-year period) because of limited time of study and lack of finance to cover accommodation costs during data collection and analysis).

## 3. Results

### 3.1. Community's Knowledge, Attitudes, and Practices (KAP) toward Tuberculosis

Out of 320 interviewees, 61.2% of them were male, whereas 38.8% were females. On the other hand, the majority of the respondents, 158 (49.4%), were found between the age of 18 and 40 years (Table 3).

Out of 320 participants on knowledge of tuberculosis, all of them claimed that they heard about TB and able to describe as tuberculosis is a contagious communicable disease. Further assessments of respondents' knowledge towards tuberculosis were shown in Table 4.

Out of 320 participants interviewed, the majority of them, 318 (99.4%), had a positive responses on seeking medical service immediately if they get clinical signs of tuberculosis. But, 242 (75.6%) disagreed with the idea that tuberculosis can be transmitted from animals to humans and vice versa (Table 5).

Multivariable ordinal logistic regression showed that there was statistically significant differences between age groups, residence, level of education, and professions (*p*value < 0.05) unlike sex which revealed insignificant association (Table 6).

### 3.2. Retrospective Data on Tuberculosis Cases

From the 51 human tuberculosis (TB) cases recorded, 32 (62.7%) were registered in Jarso Health Center, and 19 (37.3%) were nonregistered obtained through extensive searching using snowball method. Among the human tuberculosis cases, 37 (72.5%) patients were pulmonary TB positive (Table 7).

**Figure 2:**
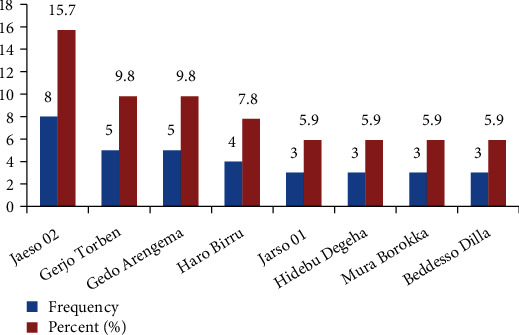
The most tuberculosis cases/prevalent villages of the Jarso District.

From this study district, the higher TB cases were obtained from Jarso 02 village eight patients (15.7%) followed by Gerjo Torben village and Gedo Arengema which were five patients (9.8%) (Figure 2). Four patients (7.8%) from Gerjo torben and Gedo Arengema, three patients (5.9%) from Beddesso Dilla, three patients (5.9%) from Haro Birru, two patients (3.9%) from Babo torben, and two patients (3.9%) from Haro Seden were searched extensively and interviewed.

### 3.3. Public Health and Economic Burden of Tuberculosis in the Jarso District

#### 3.3.1. Estimation of the Socioeconomic Burden

Control and prevention costs of TB, according to the response from the study participants, they lost two to three weeks until they go to health center for TB diagnosis before starting TB treatment which expose them to excessive out-of-pocket expenditures for informal care-seeking. For example, travelling to private pharmacy to buy simple cough remedies or pain relief before they were diagnosed.

To estimate laboratory and card costs, since biopsy diagnosis of extrapulmonary TB is not available at Jarso Health Center, ten patients were travelled to Nekemte National Clinic for only diagnosis of extrapulmonary TB, but they turned back to Jarso Health Center to obtain treatment for the disease. To diagnose extrapulmonary tuberculosis, the cost was 350 ETB per patient, but 10 patients were diagnosed with biopsy for extrapulmonary tuberculosis at Nekemte National Clinic which was computed at 4,500 ETB (128.76US$) in addition to card costs. Other ten patients were diagnosed at Nedjo Jarra Higher Clinic; the average cost of radio graphic chest X-ray was estimated at 250 ETB per patient that was computed at 3000 ETB (85.84US$) with card costs. The card costs incurred by other 32 TB patients registered in Jarso District health center were 20 ETB per patient. Therefore, the card costs for all 32 patients were 32∗20 ETB = 640 ETB (18.31US$). Although anti-TB medication was given free of charge, according to the data given from the Jarso Health Center, the average cost of 1 kit (full dose per patient) of anti-TB drugs was estimated by the government as burden to the economy of the country. These costs affect the country's economy and also affect the community indirectly by hindering the achievement of other facilities like infrastructures, hospitals, and schools.

According to the estimation, the average cost of 1 kit (full dose per patient) of anti-TB medication was about 698.04 ETB. Since the total cases of TB patients during the retrospective study including extensive searching (snowball) method were fifty-one (51) cases, the total costs of treatment for all patients were estimated at 698.04 ETB∗51 = 35,600.04 ETB (1,019.60US$). The costs of supplement drugs like multivitamins and antipains were estimated at 2,500 ETB (71.53US$). In this study, prevention costs were also estimated. Hence, according to the data from the health center, the cost of 1 vial (20 doses) of Bacillus Calmette-Guérin (BCG) infant vaccine was estimated at 96.97 ETB. When this cost is converted to 1 dose per infant, it was about 4.85 ETB. The total number of infants vaccinated during this study in one year, according to the data from the case book registration of Jarso Health Center, was 282 (two hundred eighty-two). From this data, the total cost of BCG infant vaccine given to all infants per year was calculated as 4.85 ETB∗282 = 1,367 ETB (39.11US$).

#### 3.3.2. Cost of Time Lost for Seeking Medical Treatment and Transportation Costs

To estimate the nonhealthcare costs, all the summation of transportation costs, numbers of workdays lost for seeking medical treatment, costs of transportation for extrapulmonary tuberculosis (TB) at Nekemte National private Clinic by travelling long distance of 220 km, and costs of food and bed services were assessed and expressed in monetary terms. Time lost for seeking treatment was estimated based on the total numbers of TB patients for both retrospective and snowball methods. Since for each TB patient, one contact person (caregiver) is required, for 51 TB patients, 51 contact persons were required whose time, costs of transportation and workdays were lost to give care to the patients and were assessed.

The numbers of workdays lost by the TB patients were varied based on different types of factors like severity of the disease, the strength of their immune responses to the disease, and patient delay. Although the majority of respondents (51%) answered that they lost 3months of total workdays because of their illness, it ranged from 2 months to 7 months for all patients according to the answers of respondents. Therefore, the average total workdays lost by each TB patient were 4 months. However, the total workdays lost per week were 6 days; whereas, the total workdays lost in one month were 24 days. Based on the above data, the average total workdays lost in 4 months = 4∗24 days = 96 average total workdays were lost by each TB patient. Since the total numbers of patients were forty-nine (49), the total workdays lost by all TB patients were 49^∗^96 =4,704, and average workdays were lost.

The total number of workdays lost by contact persons (caregivers) until the patient recover (feeling well) from his/her illness and can help him/herself was also varied based on the conditions of the patients. Even though the majority of respondents answered 2 months of total workday lost, it ranged from 1 month to 2 months. Therefore, the average total workdays lost by each of their caregivers were 1.5 months. Since the total workdays lost in one month were 24 days, the average total workdays lost in 1.5 months = 1.5∗24 days = 36 average workdays were lost by each of their escorts. The total numbers of their escorts were fifty-one (51) in addition to caregivers of the two dead TB patients whose workdays were also lost. Therefore, the average total workdays lost by all of the 51 escorts = 51∗36 = 1,836 average workdays were lost. Then, the average total workdays lost by all TB patients and the average total workdays lost by their escorts were added together to calculate the total workdays lost. Hence, the average total workdays lost = 4,704 + 1,836 = 6,540. However, the Ethiopian labor proclamation in its article determines the maximum daily hours of work, which states the normal hours of work shall not exceed eight hours per day [37].

In this study, the total workdays lost for seeking treatment by patients and their escorts were estimated at 6,540∗8 hrs = 52,320 hrs. Then, the normal hours of workdays lost were 52,320 hours. When 52,320 hrs is converted to workdays, it was about 2,180 workdays that were lost. The local day labor cost during the study period was 70 ETB. Therefore, the estimated economic loss from loss of workdays = 2,180 days∗70 ETB = 152,600 ETB (4,366.24US$). The other nonhealthcare costs assessed were costs of transportation per round trip; for all fifty-one TB patients and their escorts, they were estimated at 20,000 ETB (572.25US$) until the end of therapy, based on the response from respondents. Costs of food and bed services paid for 10 patients who were diagnosed at Nekemte National clinic were estimated at 15,000 ETB with their caregivers. Costs of food and bed services paid for the others 10 patients who were diagnosed at Nedjo Jarra higher clinic were estimated at 15,000 ETB with their caregivers. Costs of food and bed services paid for other 31 patients who were diagnosed and treated at Jarso District Health Center were estimated at 20,000 ETB with their caregivers. Therefore, the total costs of food and bed services were estimated at 50,000 ETB (1430.62US$). In general, the total nonhealthcare costs were the summation of the time lost while seeking treatment, cost of transportation, cost of food, and cost of bed services. Then, it was estimated at 152,600 ETB (4,366.24US$) + 20,000 ETB (572.25US$) + 50,000 ETB (1430.62US$) = 222,600 ETB (6,369.10US$). The total costs of both healthcare costs and nonhealthcare costs were estimated at 47,607.04 ETB + 222,600 ETB = 270,207.04 ETB (7,731.25US$). Only the costs of anti-TB drugs were covered by the healthcare system, but all others costs were covered by the patients and their escorts (Table 8).

#### 3.3.3. Public Health Burden Estimation

Two TB patients (3.9%) died from 51 cases at different ages due to tuberculosis.

(2) $$\begin{array}{c} \text{YLL}=\text{Number of death}{\text{s}}^{*}\text{Life expectancy at age of death},\\ \text{L}=\text{Standard Life expectancy at age at which death occurs},\\ \text{YLD}=\text{Number of case}{\text{s}}^{*}\text{D}{\text{W}}^{*}\text{L},\\ \text{DW}=\text{Disability weight},\\ \text{L}=\text{Average duration of disability}\left(\text{in years}\right). \end{array}$$

Two TB patients died because of tuberculosis at age of 41 and 49 years. The standard life expectancy at age at which death occurred for the first dead TB patient due to premature death because of tuberculosis was calculated as 66.5 − 41 years = 25.5 years. Whereas, the life expectancy at age at which death occurred for the second dead TB patients due to premature death because of tuberculosis was calculated as 66.5 − 49 years = 17.5 years. Years of life lost (YLL) = 1∗25.5 years = 25.5 years and 1∗17.5 years = 17.5 years for the first and second deceased person, respectively. Therefore, YLL = 25.5 years + 17.5 years = 43 years. Duration of disability was varying based on severity of the disease according to the response of the study participants. Therefore, it was calculated for each of the 51 TB cases, and finally, the result was multiplied by the number of cases. The duration of disability was calculated for each of the TB patients based on the severity of the disease according to the response from respondents. Then, the total result of duration of disability for all fifty-one TB cases was 7.5 years; whereas, the disability weight of TB is 0.271. Therefore, YLD = 7.5 years∗0.271 = 2.03 years. Therefore, the total DALYs lost due to premature death because of tuberculosis = 43 years + 2.03 years = 45.03 years. The total estimated economic and public health burden of tuberculosis in Jarso District was illustrated in Table 8.

## 4. Discussion

The socioeconomic characteristics of this study participants showed that most of them (196 (61.2%)) were males; whereas, the rest (124 (38.8%) were female respondents. The ages of respondents were divided into three categories: 18-40, 41-60, and > 60 years to gather information according to their age categories. Accordingly, from the sampled interviewees, 158 (49.4%) were between 18 and 40 years old, 146 (45.6%) were between 41 and 60 years old, and 16 (5.0%) were > 60 years old. The majority of the respondents were illiterates (those who cannot write and read) accounted for 140 (43.7%); whereas, 96 (30%), 45 (14.1%), and 39 (12.2%) of the respondents attended primary, secondary, and tertiary levels of education, respectively. The educational level and age category of this study participant are very important in implementing awareness creation campaigns and behavioral improvement in the communities. Farmers held a higher percentage, 128 (40.0%) (Table 3).

The current study was conducted to assess the KAP of the community towards tuberculosis and to estimate the public health and financial burden of the disease in Jarso District. Out of 320 interviewees, all of them heard about tuberculosis, and most of the respondents defined tuberculosis as a disease of the lung that comes from cold air (Table 4). This result is nearly in line with the finding of Bati et al. [38], conducted in Gambella Region, Itang Special District, who reported that most of the community's members have information about tuberculosis and similar to the finding of Bashorun et al. [39], conducted in Gambia, who reported that most participants indicated they had heard about tuberculosis. All of the respondents agreed that tuberculosis is a contagious communicable disease; however, only 25 (7.8%) of the study participants correctly answered the cause of TB as being germ/bacteria, and predisposing factors like cold air, alcohol consumption, smoking, and shortage of food were frequently mentioned as the cause of tuberculosis by the present study participants (Table 4), which was similar to the study conducted in Afar and North Ethiopia by Yimer et al. [40]. On the other hand, 210 (65.6%) of the participants disagree that raw animal food products are source of TB which implies that they are at risk of contracting TB from consumption of raw animal products.

The majority of respondents, 242 (75.6%), disagreed with the idea that tuberculosis can be transmitted from animals to humans and vice versa, while a few of them, 78 (24.4%), agreed as TB is zoonotic. Many of the participants, 214 (66.9%), disagreed that pasteurization of milk before consumption prevents tuberculosis; whereas, a few of them, 106 (33.1%), agreed as it prevents TB (Table 5). This finding was consistent with the finding of Bihon et al., [41] conducted in and around Gondar town, who reported that the highest proportion of the respondents did not consider the consumption of the raw animal products (milk and meat) to pose a risk for exposure to bovine tuberculosis.

Multivariable ordinal logistic regression analysis showed that there was statistically significant difference between age groups (OR = 105.81 (95% CI: 1.28-8715.94)); (*P* = 0.038) (96.48 (95% CI: 1.16-8020.16)) (*P* = 0.043), where the younger age groups (18-40 years) has shown 105.8 times more odds of knowledge towards the science of tuberculosis than the older counterpart (>60 years). Similarly, respondents in the age range of 41-60 years had shown 96.5 times more likely to have knowledge of tuberculosis than the older ones (Table 6). This finding was consistent with the report of Bashorun et al. [39] conducted in Gambia, who reported that age groups of 35-64 had good TB knowledge, a favorable attitude, and good practices towards TB, compared to ≥65 years. Urban participants had 2.24 times more odds of knowledge towards tuberculosis (OR =2.24 (95% CI: 1.33-15.33, *P* < 0.023)), compared to rural participants (Table 6). This finding was consistent with the report of Oladele et al. [42], conducted in Nigeria, who reported that higher proportion of urban respondents had better knowledge of TB compared to rural areas. However, this result disagreed with the finding of Bashorun et al. [39], conducted in Gambia, who reported that TB awareness was higher in rural compared to the urban residents.

Tertiary level education participants had 9395.08 times more odds of knowledge towards the science of tuberculosis (OR = 9395.08, 95% CI: 212.62-415132, *P* < 0.001), compared to illiterates. This finding was similar with the finding of Luba et al. [43], conducted in Lesotho, who reported the study participants who had higher educational levels were 6.26 more likely to be aware of TB than those who had no education. Secondary level education participants had 2051.3 times more odds of knowledge towards the science of tuberculosis (OR = 2051.3, 95% CI: 135.27-31107.4, *P* < 0.001), compared to illiterates. Primary levels of education participants had 236.63 times more odds of knowledge of the science of tuberculosis (OR = 236.63, 95% CI: 42.96-1303.43, *P* < 0.001), compared to illiterates. Health professionals had 34.17 times more odds of knowledge towards the science of tuberculosis (OR = 34.17, 95% CI: 1.79-1300.2, *P* = 0.015), compared to farmer participants. Similarly, teachers had 32.53 times more odds of knowledge towards the science of tuberculosis (OR = 32.53, 95% CI: 1.15-33.9, *P* = 0.026), compared to farmer participants (Table 6).

In the current study, the higher TB record was in rural communities 40 (78.4%) compared to the urban communities. This may be because of inaccessibility of public health education and sanitary practices for the rural community than the urban community which is one of the predisposing factors for transmission of the disease (Table 7). This finding disagrees with the study conducted by Datiko et al., [44] in Adama City, who reported that the higher tuberculosis cases registered at health facilities came from Adama City compared to other catchment areas. This may be because of the frequent consumption of raw animal products and by-products of the urban communities than the poor rural communities which was another risk factor for the transmission of tuberculosis from animals to humans.

Out of 51 human tuberculosis, 37 (72.5%) were pulmonary positive due to mostly mode of transmission of tuberculosis is through droplet nuclei (airborne particles) reaching the alveoli of the lungs, 5 (9.8%) were pulmonary negative, and 9 (17.6%) were extrapulmonary tuberculosis (Table 7). This finding was similar with the finding of the Asres et al. [45], who reported that 79.7% of the patients were pulmonary TB in South West Ethiopia. All of the patients were treated with anti-TB drugs like rifampicin ®, isoniazid (H), pyrazinamide (Z), and ethambutol (E). The most tuberculosis prevalent villages of the Jarso District were Jarso 02 village 8 (15.7%), Gedo Arengema 5 (9.8%), Gerjo torben 5 (9.8%), Haro Birru 4 (7.8%), Jarso 01, Mura Borokka, Hidebu Degeha, and Beddesso Dilla villages 3 (5.9%) (Figure 2) and subsequently were reached and interviewed; whereas, 17 (33.3%) patients were interviewed using telephone since they were located in the remote areas of the district as well as found in the scattered patterns.

In the present study, the magnitude of tuberculosis in terms financial burden aspect was estimated (Table 8). The economic burden was estimated with the consideration of direct and indirect costs. The direct healthcare costs were estimated with costs of diagnosis, card costs, costs of anti-TB medications, costs of other supplementary drugs like multivitamins and pain killers, and costs of Bacillus Calmette-Guérin (BCG) infant vaccine; whereas, the direct nonhealth costs that were estimated were costs of transportations and costs of food and accommodation; whereas, the average loss of work time for seeking treatment was considered as indirect nonhealthcare costs. The number of workdays lost due to the patient and for the escort and wage missed due to seeking treatment was quantified to be 152,600.00 ETB (4,366.24US$).

The healthcare costs and nonhealthcare costs were estimated at 270,207.04 ETB (7,731.25US$) (Table 8). This is nearly in line with the finding of the study conducted in South West Ethiopia by Asres et al. [45], who assessed the total direct and indirect costs incurred by patients for care-seeking diagnosis and treatment amount to a median interquartile range (IQR) of US$201.48 (136.70–318.94). However, this finding was lower compared to Tiemersma and Hafidz [46], conducted in India, who reported that a study of MDR-TB patient costs in March 2013 indicated that the average total out-of-pocket cost for an MDR-TB patient to get diagnosis and treatment was 1,341US$, and each patient lost, on average, 293 US$ of income due to time spent seeking and receiving care (excluding income losses due purely to inability to work).

To assess the public health burden for the two deceased people, the disability-adjusted life years (DALYs) were calculated by summing the number of years of life lost due to mortality (YLL) and the number of years lived with a disability due to morbidity (YLD) [47]. The standard life expectancy of age at which death occurred for the first deceased TB patients due to premature death because of tuberculosis was calculated as 66.5 − 41 years = 25.5 years. Whereas, the life expectancy of age at which death occurred for the second deceased TB patient due to premature death because of tuberculosis was calculated as 66.5 − 49 years = 17.5 years. Years of life lost (YLL) = 1∗25.5 years = 25.5 years and 1∗17.5 years for the first dead person and the second dead person, respectively. Therefore, YLL = 25.5 years + 17.5 years = 43 years. The total result of duration of disability for all fifty-one TB cases was 7.5 years; whereas, the disability weight of TB is 0.271. Therefore, YLD = 7.5 years∗0.271 = 2.03 years. Therefore, the total DALYs lost due to premature death because of tuberculosis = 43 years + 2.03 years = 45.03 years (Table 8). This finding was very low when compared to other findings conducted in Texas in which a total of 1189 DALYs were lost because of tuberculosis [48]. From the total DALYs lost, the largest contribution to the public health burden was due to YLLs 43 (95.5%) rather than YLD.

## 5. Conclusion

Based on KAP assessment, all participants of the district heard about tuberculosis. However, most of the communities of the study district were unaware of the cause of TB and its sources of infection. From the retrospective study, people whose age groups between 40 and 60 years have higher percentage when compared to other age groups. This study showed that the estimated economic burden within one year was 7,731.25 US$; whereas, the estimated public health burden was 45.03 DALYs or years of healthy life lost which was burden to the district. This finding highlights the need for health education efforts to strengthen accurate information dissemination to promote sound TB knowledge, attitudes, and practices in the community as well as the inauguration of one health approach between veterinary and public health disciplines to work in collaboration to combat this zoonotic catastrophe. Further research would be vital to study specific species of Mycobacterium found in the study area and long year's economic analysis and public health burden to give specific direction for implementation of the prevention of the disease.

## Figures and Tables

**Table 1:** The study area description of Jarso District.

Description	Jarso District
Distance from Addis Ababa and Gimbi	543 km and 103, respectively
Agroecology	80% midland and 20% low land
Altitude	1840 to 1860 m.a.s.l
Mean annual rainfall	900 mm to 1400 mm
Mean maximum and minimum temperature	25°C and 18°C, respectively
Human populations	69,636
Male	34,122
Female	35,514
The total livestock populations	224,958
Cattle	98,756
Sheep	33,004
Goats	21,570
Equines	5,518
Pet animals	5,630
Chickens	60,480

Source: Jarso District Agricultural Office, 2021 (unpublished).

**Table 2:** Cost classification to estimate burden of tuberculosis.

Direct costs		Indirect costs
Healthcare costs	Nonhealthcare costs	Mortality (DALYs) Morbidity (DALYs)
Diagnosis	Transportation	
Vaccination	Loss of time while moving to health center	
Wound care and disinfection	Loss of time of their family (relatives) to help the patient	
Antibiotics	Loss of time while being in bed	

**Table 3:** Sociodemographic profiles of respondents from Jarso District.

Characteristics	District (*N* = 320)	Residence (320)		Total
	Jarso	Urban	Rural	
Sex	Male	89 (27.8%)	107 (33.4%)	196 (61.2%)
	Female	62 (19.4%)	62 (19.4%)	124 (38.8%)

Age (years)	18-40	88 (27.5%)	70 (21.9%)	158 (49.4%)
	41-60	57 (17.8%)	89 (27.8%)	146 (45.6%)
	>60	6 (1.9%)	10 (3.1%)	16 (5.0%)

Educational status	Illiterate	35 (10.9%)	105 (32.8%)	140 (43.7%)
	Primary	73 (22.8%)	23 (7.2%)	96 (30.0%)
	Secondary	23 (7.2%)	22 (6.9%)	45 (14.1%)
	Tertiary	20 (6.2%)	19 (5.9%)	39 (12.2%)

Occupation	Farmers	16 (31.4%)	14 (27.5%)	128 (40%)
	H/professionals	2 (0.6%)	28 (8.8%)	30 (9.4%)
	Teacher	15 (4.7%)	8 (4.7%)	23 (7.2%)
	Merchants	37 (11.6%)	2 (0.6%)	39 (12.2%)
	Others	82 (25.6%)	18 (5.6%)	100 (31.2%)

H: health; Others: students, jobless, and butchers; illiterate (cannot write and read).

**Table 4:** Knowledge of respondents towards tuberculosis regarding cause, sources, symptoms, and clinical forms of the disease.

Knowledge of respondents toward tuberculosis (*N* = 320)	Categories	Frequency (no)	Percentage (%)
Heard about tuberculosis	Agree	320	100

Mycobacteria are the cause of TB	Agree	25	7.8
	Disagree	295	92.2

Raw animals' food products are sources of TB	Agree	110	34.4
	Disagree	210	65.6

Coughing for two/above two weeks, night sweating, loss of appetite, and loss of body weight are clinical signs of TB	Agree	299	93.4
	Disagree	21	6.6

Pulmonary and extrapulmonary types of TB can occur in family members	Agree	189	59.1
	Disagree	131	40.9

Young people are more affected by tuberculosis	Agree	38	11.9
	Disagree	282	88.1

Tuberculosis is a contagious communicable disease	Agree	320	100

**Table 5:** Attitude and practices of the study participants towards tuberculosis (*N* = 320).

Attitude and practices of respondents (*N* = 320)	Categories	Frequency (no)	Percentage (%)
Anybody exposed to TB should seek medical service	Agree	318	99.4
	Disagree	2	0.6

Tuberculosis is a preventable disease	Agree	249	77.8
	Disagree	71	22.2

Tuberculosis is prevented by vaccination	Agree	146	45.6
	Disagree	174	54.4

Pasteurization of milk before consumption prevents tuberculosis	Agree	106	33.1
	Disagree	214	66.9

Opening windows regularly prevents tuberculosis	Agree	158	49.4
	Disagree	162	50.6

Tuberculosis can be transmitted from animals to humans	Agree	78	24.4
	Disagree	242	75.6

**Table 6:** Univariable and multivariable logistic regression analysis of the KAP of community of Jarso District, West Ethiopia.

Variables	*N* (%)	Univariable analysis			Multivariable analysis		
		OR	95% CI	*P* value	AOR	95% CI	*P* value
*Age category*							
18-40	158 (49.4)	85.22	10.69–679	<0.001	105.81	1.28–8715.94	0.038
41-60	146 (45.6)	12.86	1.65-100.46	0.015	96.48	1.16–8020.16	0.043
>60 (ref)	16 (5.0)						
*Sex*							
Male	196 (61.2)	1.86	1.14-3.03	0.012	2.35	0.84–6.57	0.105
Female (ref)	124 (38.8)						
*Residence*							
Urban	151 (47.2)	3.89	2.35-6.44	<0.001	2.24	1.33–15.33	0.023
Rural (ref)	169 (52.8)						
*Educational*							
Illiteracy (ref)	76 (23.8)						
Write and read	64 (20.0)	37.49	10.63-132.23	<0.001	61.24	13.77-272.43	<0.001
Primary	45 (14.1)	137.52	33.66-561.85	<0.001	236.63	42.96–1303.43	<0.001
Secondary	39 (12.2)	912	91.65-9075.12	<0.001	2051.30	135.27–31107.4	<0.001
Tertiary	96 (30.0)	1203.33	196.06-7385.62	<0.001	9395.08	212.62–415132	<0.001
*Occupation*							
Farmer (ref)	128 (40.0)						
H/Prfns.	30 (9.4)	9.33	2.03–42.80	<0.001	34.17	1.79-1300.2	0.015
Teacher	23 (7.2)	30.66	4.01–234.1	<0.001	32.53	1.10-33.90	0.026
Merchant	39 (12.2)	1.7	0.84–3.47	0.142	5.27	0.10-255.56	0.686
Others	100 (31.2)	6.999	3.22–11.16	0.020	1.83	0.88-1361.02	0.401

H/Prfns: health professionals; Ref: reference cell; Others: students, jobless, and butchers.

**Table 7:** Descriptions of retrospective data of TB patients between September 2019 and 2020.

Descriptions	Categories	Frequency	Percentage (%)
Age (in years)	0-17	4	7.8
	18-40	24	47.0
	41-60	19	37.3
	>60	4	7.8

Sex	Male	24	47.0
	Female	27	52.9

Residence	Urban	11	21.6
	Rural	40	78.4

Registration status of TB	^∗^Registered	32	62.7
	^∗^Nonregistered	19	37.3

Place of diagnosis	Public health center	31	60.8
	Privet clinics	20	39.2

Type of TB	^∗^Pulmonary +ve	37	72.5
	^∗^Pulmonary^_^ve	5	9.8
	Extrapulmonary	9	17.6

^∗^Outcome of Rx	Cured	49	96.1
	Death	2	3.9

^∗^Registered: registered in Jarso Health Center, ^∗^Nonregistered: searched extensively using snowball methods, ^∗^Outcome of Rx: the result obtained after treatment, ^∗^Pulmonary +ve: bacteriologically confirmed, and ^∗^Pulmonary^_^ve: bacteriologically unconfirmed.

**Table 8:** Summary of estimated economic and public health burdens of tuberculosis in Jarso District.

Funding source	Cost categories	Source of data	ETB	US$
	Direct costs			
TB patients and escorts	Transportation	Patient interview	20,000.00	572.25
TB patients	Biopsy & X-ray dx	Patient interview	8,140.00	232.90
TB patients	Supplement drugs	Patient interview	2,500.00	71.53
TB patients and escorts	Food and lodging	Patient interview	50,000.00	1,430.62
	Indirect costs			
TB patients and escorts	Loss of workdays	Patient interview	152,600.00	4,366.24
	Direct health costs			
Public health system	Anti-TB medication	Medical record	35,600.04	1,019.60
	BCG vaccine	Medical record	1,367.00	39.11
Total economic loss			270,207.04	7,731.25
Total DALYs lost or years of healthy life lost			45.03 DALYs	

## Data Availability

Datasets used and/or analyzed during the current study are available from the corresponding author on reasonable request.
